# Developing Artefact Removal Algorithms to Process Data from a Microwave Imaging Device for Haemorrhagic Stroke Detection

**DOI:** 10.3390/s20195545

**Published:** 2020-09-28

**Authors:** Behnaz Sohani, James Puttock, Banafsheh Khalesi, Navid Ghavami, Mohammad Ghavami, Sandra Dudley, Gianluigi Tiberi

**Affiliations:** 1 School of Engineering, London South Bank University, London SE1 0AA, UK; puttockj@lsbu.ac.uk (J.P.); khalesib@lsbu.ac.uk (B.K.); ghavamim@lsbu.ac.uk (M.G.); dudleyms@lsbu.ac.uk (S.D.); g.tiberi@iet.unipi.it (G.T.); 2 UBT-Umbria Bioengineering Technologies, Spin off of University of Perugia, 06081 Assisi, Italy; navid.ghavami@kcl.ac.uk

**Keywords:** microwave imaging, brain stroke detection, portable medical devices, UWB imaging, artefact removal methods, Huygens principle

## Abstract

In this paper, we present an investigation of different artefact removal methods for ultra-wideband Microwave Imaging (MWI) to evaluate and quantify current methods in a real environment through measurements using an MWI device. The MWI device measures the scattered signals in a multi-bistatic fashion and employs an imaging procedure based on Huygens principle. A simple two-layered phantom mimicking human head tissue is realised, applying a cylindrically shaped inclusion to emulate brain haemorrhage. Detection has been successfully achieved using the superimposition of five transmitter triplet positions, after applying different artefact removal methods, with the inclusion positioned at 0${}^{{}^{\circ}}$, 90${}^{{}^{\circ}}$, 180${}^{{}^{\circ}}$, and 270${}^{{}^{\circ}}$. The different artifact removal methods have been proposed for comparison to improve the stroke detection process. To provide a valid comparison between these methods, image quantification metrics are presented. An “ideal/reference” image is used to compare the artefact removal methods. Moreover, the quantification of artefact removal procedures through measurements using MWI device is performed.

## 1. Introduction

Several imaging techniques are regularly employed in the process of brain stroke diagnosis, notably magnetic resonance imaging (MRI) and computed tomography (CT). The Microwave Imaging (MWI) procedure is based on the scattering of electromagnetic waves. It is capable of fulfilling several essential requirements such as being fast, portable (in consequence of the dimensions of the devices) and non-ionising in nature, and needing reduced intensity to achieve imaging (with the intensity similar to that applied in cellular phones). These significant features of MWI not only offer the possibility of an appropriate and safe imaging modality for repeated medical applications, but also allow for the construction of relevant devices for use at small medical centres for both monitoring and detection of a variety of cardiovascular diseases. MWI relies on significant differences in dielectric properties consisting of the electric permittivity and conductivity between healthy and stroke tissues [1]. These differences provide an opportunity for a functional map of the examined anatomical region to be achieved. A haemorrhagic stroke inside a healthy human head illuminated by an electromagnetic field transmitted by a dedicated antenna generates a scattered field, which, if properly processed and measured, may be applied to detect and locate the brain haemorrhage itself. In [2], the dielectric properties of human tissues have been widely discussed and measured. A number of MWI prototypes and devices for brain stroke diagnostics have been offered recently [3,4,5].

Amongst the substantive research in medical MWI prototypes, some experimental schemes have been developed for the detection of brain abnormalities, specifically for brain stroke detection and monitoring. Two prototypes (which have been recently tested on the human body) are the most prominent types amongst MWI prototypes. The first is the “Stroke-finder”, which was developed by Medfield Diagnostics at Chalmers University [3,4]. The second is the “BRIM G2” developed for brain scanning at EMTensor [5]. The Medfield Diagnostics device is a prototype used for the detection, classification and discrimination between two sorts of intracranial bleedings (brain haemorrhage and brain ischemia) in order to diagnose patients at the early onset of stroke. An early diagnosis of stroke using this device can be performed on the basis of an automated classification, achieved by making a comparison between the measurements and a database which has been constructed from collected data from examined patients. The device is categorised according to its ordinary and compact hardware and consists of 12 transmitting/receiving triangular printed antennas fixed on a support which is capable of being adapted to the patient’s head (i.e., helmet-shaped). Although, the early clinical trials have been represented in [4], it is worth noting that this device cannot provide images. The “BRIM G2” is developed to exhibit brain stroke tomography. This is a high complexity device comprised of 177 radiating elements (rectangular ceramic-loaded antennas operating at 1 GHz) [5]. Although this prototype successfully detected stroke and offered good experimental reconstruction results for brain monitoring, it suffers from some considerable disadvantages. The configuration of 177 radiating elements can significantly increase both the size and cost of this device. Therefore, the EMTensor has several limitations. Moreover, to perform the image reconstruction, there is a need for processing a significant quantity of measured data and finding a solution to a nonlinear and ill-posed inverse scattering problem (as a result of using the microwave tomography (MWT) system). The need to solve inverse problems can significantly increase the computational overhead, elaboration times and possibly cause false solutions. The configuration of brain imaging MWT scanner has been studied in [6] and [7] for optimising the design of antenna array and establishing the appropriate frequencies and properties of the coupling medium. In a MWT procedure, the array antenna transmits electromagnetic waves and the object scatters these waves (the waves penetrate into the tissue). Then, the receiver will receive and collect the scattered field [8]. The MWT algorithm is applied to reconstruct a map of the dielectric properties relevant to the imaged area to locate an object with unidentified dielectric properties by finding a solution to a nonlinear and ill-posed inverse scattering problem [9]. In [10], it is noted that the brain haemorrhage changes the tissues’ dielectric properties. Brain haemorrhage causes an increase of up to 20% in the dielectric properties with respect to the normal white/grey matter dielectric properties [11].

In response to the mentioned limitations of MWT systems and the medical necessities of brain stroke detection, a robust procedure on the basis of the Huygens principle (HP) method has previously been developed for haemorrhagic brain stroke detection using MWI techniques [12]. Head imaging has also been investigated through phantom measurements inside an anechoic chamber, using two antennas in free space and the imaging procedure based on HP [12]. Since the results coming from measurements inside the anechoic chamber are not representative of a realistic medical device scenario, and there is a medical requirement for a portable brain stroke imaging device, we have come to the decision to apply different signal pre-processing methods to the imaging results collected from a portable MWI device for brain haemorrhage imaging. Signal pre-processing methods are predominantly used to remove artefacts, i.e., transmitter image and the reflections of the layers. If the artefact is not removed fully, the inclusions (e.g., the strokes or tumours) will be masked, and thus the detection will not be accurate. Recently, a portable MWI device, which operates in free space with two azimuthally rotating antennas, has been developed and employed for breast cancer detection [13]. More specifically, the device consists of two antennas which rotate around the breast to collect signals in a multi-bistatic fashion. The purpose of this paper is to present an investigation on different artefact removal methods for ultra-wideband (UWB) MWI in order to evaluate and quantify these methods in a real environment through measurements using an MWI device. In this manuscript, a simple multi-layered phantom mimicking human head tissue is realised, applying a millimetric cylindrically shaped inclusion to emulate brain haemorrhage. Moreover, the quantification of artefact removal procedures through measurements using the MWI device is performed. The portable MWI device, which was initially constructed for breast cancer detection, measures the scattered signals in a multi-bistatic fashion and employs an imaging procedure based on HP.

The remainder of this paper is organised as follows: Section 2 describes HP based imaging procedures, different artefact removal methods and the quantification of these artefact removal procedures through MWI device, the MWI device and methodology for brain phantom characterisation. In Section 3, we present the reconstruction results for haemorrhage mimicking target through measurements using MWI device. Section 4 summarises our findings and discusses the obtained results. Finally, Section 5 concludes the results and explains the future prospects towards artefact removal quantification using an MWI device for brain haemorrhage imaging.

## 2. Materials and Methods

### 2.1. Description of HP Based Imaging Procedure

It has been discussed in a study on breast cancer detection [14] that microwave technology relies on detecting the contrast at the interface between two tissues with differing dielectric properties (between healthy and malignant tissues). UWB technology has recently been applied to medical applications for detection and monitoring purposes. The use of UWB offers numerous benefits, for instance high resolution (owing to the high bandwidth spectrum) in order to indicate the inclusions in resulting images, high capacity and reliability, and low power transmission. The same principle has been applied to determine the contrast between blood and brain matter to identify stroke in head mimicking phantoms [15]. In this paper, an algorithm based on HP is applied to forward propagate the waves [16]. The use of HP removes the necessity for having to solve complex inverse problems. The scattered electric field *E* is achieved through Equation (1) by summing the signals $S21_{np,\mathrm{tx}}$ collected at the points $rx_{\mathrm{np}}$ displaced along a circular surface having radius $a_{0}$, where np is the number of receiving points (from 1 to $N_{\mathrm{PT}}$):

(1) $$E_{\mathrm{HP},2D}^{\mathrm{rcstr}}\left(\rho ,\phi ;{\mathrm{tx}}_{m};f\right)=\Delta_{s}\sum_{np=1}^{N_{\mathrm{PT}}}S21_{np,{\mathrm{tx}}_{m}}G\left(k_{1}|{\vec{\rho }}_{\mathrm{np}}-\vec{\rho }|\right)$$

In the above equation, $\vec{\rho }$ ≡ (ρ,ϕ) is the observation point; $k_{1}$ represents the wave number for the media in the imaging zone; $\Delta_{s}$ is the spatial sampling (representing the distance between two adjacent receiving points that can be calculated by Equation (2), and ${\mathrm{tx}}_{m}$ indicates the number of transmitting antennas operating at frequency *f*. The “reconstructed” internal field has been indicated by the string “rcstr”, whilst the string HP indicates that Huygens based procedure will be employed in Equation (1). S21 is the parameter representing the complex transfer function from the transmitting antenna to the receiving antenna.

(2) $$\Delta_{s}=\frac{2\pi a_{0}}{N_{\mathrm{PT}}}$$

In Equation (1), the Green’s function *G* is applied to propagate the field using Equation (3);

(3) $$G\left(k_{1}|{\vec{\rho }}_{\mathrm{np}}-\vec{\rho }|\right)=\frac{1}{4\pi |{\vec{\rho }}_{\mathrm{np}}-\vec{\rho }|}e^{-jk_{1}|{\vec{\rho }}_{\mathrm{np}}-\vec{\rho }|}$$

The proposed HP-based procedure differs from the Kirchhoff migration method whose algorithms usually perform time reversal and back-propagation to find the phase, that is, time, traces. Conversely, here we are not interested in finding the phase traces: in fact, we use HP with the aim of reconstructing the field. More details can be found in [17]. It should be pointed out that the reconstructed electric field depends on both illuminating source and frequency. If we use $N_{f}$ frequencies $f_{i}$, then the intensity of the final image *I* can be obtained mathematically using Equation (4).

(4) $$I\left(\rho ,\phi \right)=\sum_{i=1}^{N_{f}}{\left|E_{\mathrm{HP}}^{\mathrm{rcstr}}\left(\rho ,\phi ;{\mathrm{tx}}_{m};f_{i}\right)\right|}^{2}$$

To remove artefacts, transmitter image and the reflections of the layers, signal pre-processing methods are employed. An accurate detection will not be feasible to obtain when the artefact is not totally eliminated and the inclusions (e.g., the strokes) are masked. In this paper, we have applied different artefact removal methods with the data from the measurements in order to find the best method of artefact removal, specifically for the detection of brain haemorrhage.

### 2.2. Artefact Removal Methods

- Ideal Artefact Removal Method

In this approach of artefact removal, two sets of experimental measurements are performed for the imaging scenario; First, the “no target” scenario, for a phantom with no target. Secondly, the “target” scenario a repeat of the procedure using a phantom with a millimetric cylindrically shaped inclusion to emulate a haemorrhagic brain stroke. The inclusion will be indicated in the image obtained through Equation (4), by subtracting the recorded S21 of the “no target” scenario from that of the “target” scenario “$S21_{np,tx_{m}}^{\mathrm{rcstr},\mathrm{target}}-S21_{np,tx_{m}}^{\mathrm{rcstr},\mathrm{notarget}}$”. This expression can be used in Equation (1). An “ideal” case is then generated to prove the concept of the technology. This would mean that the “ideal” image method could be used as a reference for comparison with the resulting images from other artefact removal methods. This will be presented in the next section.

It is important to point out that, when dealing with a real scenario, there is no possibility of applying this artefact removal method to medical imaging. We apply this method to show the strong feasibility of detecting the brain haemorrhage in an ideal way. Moreover, one of the efforts of the current paper is to find an algorithm which generates as close of a match as possible to the ideal result. Applying several different artefact removal algorithms has the potential to vary the resulting image. Therefore, it is not feasible to test the algorithm in situations where the ideal response is not calculated or known (i.e., measured data from a real human head with a brain stroke). Hence, in clinical trials this artefact removal method cannot be helpful and effective.

- Rotation Subtraction (RS) Artefact Removal Method

The rotation subtraction strategy has been performed for artefact removal by employing a setup to replicate a signal from two transmitters, which have been positioned 4.5${}^{{}^{\circ}}$ apart on the perimeter of the cylinder. In the rotation subtraction method [18,19], artefacts are eliminated by subtracting two measurements collected using two slightly displaced transmitting positions. The RS strategy is described mathematically as “$S21_{np,tx_{m}}-S21_{np,tx_{m+1}}$”. This expression can be used in Equation (1). The current procedure has been implemented by subtracting the transmitting position m+1 from the transmitting position *m*, where *m* and m+1 correspond to the same set of recorded measurements.

- Local Average Subtraction (LAS) Artefact Removal Method

In this approach of artefact removal [19], we endeavour to successfully remove the artefact by using the average values of the complex S21, which have been received from many transmitting antennas. These transmitting antennas are positioned 4.5${}^{{}^{\circ}}$ apart from each other. We apply the subtraction between received signals $S21_{np,{\mathrm{tx}}_{m}}$ and the mean of received signals $avg\{S21_{np,{\mathrm{tx}}_{m}}\}$ mathematically through “$S21_{np,tx_{m}}-avg\left\{S21_{np,tx_{m}}\right\}$”. This expression can be employed in Equation (1).

- Differential Symmetric Receiver (DSR) Type Artefact Removal Method

The medium we eventually will be using (the brain) has a natural symmetry. By exploiting the (eventual) object symmetry, it may be possible to apply another artefact removal method using the difference between the receivers placed symmetrically opposite. This method is initially derived from the literature of [20,21,22] used by Mustafa et al. In the DSR Type method, artefacts are removed by performing the subtraction between each receiver value from its symmetrically opposite receiver. The artefact removal can be obtained mathematically through DSR method using the following equation:

(5) $$S\left(\rho ,\phi ;N_{\mathrm{TX}}\right)=S21_{np,tx_{m}}\left(\rho ,\phi ;N_{\mathrm{TX}}\right)-S21_{np,tx_{m}}\left(\rho ,N_{\mathrm{PT}}+2-\phi ;N_{\mathrm{TX}}\right)$$

$$\mathrm{for}\;\phi =1\;\mathrm{to}\;N_{\mathrm{PT}}\;\mathrm{with}\;N_{\mathrm{PT}}+1=\frac{N_{\mathrm{PT}}}{2}+1\;\mathrm{for}\;\phi =1\;\mathrm{and}$$

$$\frac{N_{\mathrm{PT}}}{2}+1=1\;\mathrm{for}\;\phi =1\;\mathrm{for}\;\phi =\frac{N_{\mathrm{PT}}}{2}+1$$

where $N_{\mathrm{PT}}$ is the maximum number of receiving antennas, $N_{\mathrm{TX}}$ is an index to indicate the maximum transmitting positions and $S21\left(\mathrm{np},tx_{m}\right)$ is the original recorded complex S21. This creates a Differential (Symmetric Receiver Type) matrix *S*. The AS or RS method is then applied to this matrix.

- Summed Symmetric Differential (SSD)

The DSR method mentioned above is based on the natural symmetry of some media (e.g., the brain), across the left and right halves. It is worthwhile pointing out that the images which have used the symmetric method may contain mirrored artefacts. To eliminate the mirrored section, SSD could be successful. The ellipsoidal shape of the human head has a distinct left-right line of symmetry. The front-back sections of the brain also contain similar densities of tissue. Whilst not absolutely symmetrical, the resemblance in shape and density could be utilised to offer an artefact removal method by summing a differential matrix formed from the left-right differential and a second matrix formed from a front-back differential. Hence, this can provide a more intense peak at the area of inclusion, and subsequently the mirrored artefacts will have a reduced intensity. A differential matrix *S* is constructed the same way as before by applying Equation (5). A second matrix *R* is constructed across the front-back receivers and defined as follows:

(6) $$R\left(\rho ,\phi ;N_{\mathrm{TX}}\right)=S21_{\mathrm{np},tx_{m}}\left(\rho ,\phi ;N_{\mathrm{TX}}\right)-S21_{\mathrm{np},tx_{m}}\left(\rho ,\frac{N_{\mathrm{PT}}}{2}+2-\phi ;N_{\mathrm{TX}}\right)$$

$$\mathrm{for}\;\phi =1\;\mathrm{to}\;\frac{N_{\mathrm{PT}}}{2}+1$$

$$\mathrm{with}\;\frac{N_{\mathrm{PT}}}{2}+2-\phi =\frac{3N_{\mathrm{PT}}}{4}+1\;\mathrm{for}\;\phi =\frac{N_{\mathrm{PT}}}{4}+1$$

(7) $$R\left(\rho ,\phi ;N_{TX}\right)=S21_{\mathrm{np},tx_{m}}\left(\rho ,\phi ;N_{\mathrm{TX}}\right)-S21_{\mathrm{np},tx_{m}}\left(\rho ,\frac{3N_{\mathrm{PT}}}{2}+2-\phi ;N_{\mathrm{TX}}\right)$$

$$\mathrm{for}\;\phi =\frac{N_{\mathrm{PT}}}{2}+2\;\mathrm{to}\;N_{\mathrm{PT}}$$

$$\mathrm{with}\;\frac{3N_{\mathrm{PT}}}{2}+2-\phi =\frac{3N_{\mathrm{PT}}}{4}+1\;\mathrm{for}\;\phi =\frac{3N_{\mathrm{PT}}}{4}+1$$

Then the combined matrix *C* is composed of summing matrices *S* and *R* and used as a Differential (Summed Symmetric Receiver Type) matrix. The AS or RS method is then applied to this matrix. 

### 2.3. Image Quantification Metrics

Imaging performance has been investigated using image quantification. As a portion of this research, the calculation of certain parameters will be required to quantify the imaging’s detection capabilities, compare the proposed artefact removal methods, and provide a quantifiable measurement system for comparing images. There are several metrics used for quantifying stroke detection capability.

Based on two scenarios discussed in this paper (the “no target” scenario and the “target” scenario), we introduced the calculation of six metrics which allow us to perform a quantification of detection accuracy. These metrics are categorised into those that rely on a reference image called “ideal” image and those independent of that, and are comprised of Area Difference (ArD) index, Polyshape Construction, Centroid Difference (CD), Signal-to-Noise Ratio (SNR), Signal-to-Clutter ratio (S/C) and Structural Similarity Index Metric (SSIM). Amongst these, SSIM, ArD, and CD are dependent on an “ideal” image.

For the purposes of this experiment, an “ideal” image has been considered as shown in the results section. Further details with reference to these metrics are explained below.

- Polyshape Construction

When evaluating an image, the inclusion in the “ideal” image is approximately made up of the normalised values which are over 75% of the maximum value. To evaluate the shape of the inclusion, the image is adjusted through expanding the values greater than 0.75 to 1 and enforcing to 0 the values less than 0.75. By applying MATLAB’s polyboundary and polyshape functions, the resulting shape can be achieved [23].

- Area Difference (ArD)

Having set a threshold and defined two areas as the “target” area and the “background” area, a comparison can then be drawn between the size of the target area for an “ideal” image and that of the test image. Mathematically speaking, it can be obtained through the following equation:

(8) $$\mathrm{Area}\;\mathrm{Difference}=|\frac{N_{\mathrm{Experiment}(\mathrm{test})}-N_{\mathrm{Ideal}}}{\mathrm{Image}\;\mathrm{Matrix}\;\mathrm{Size}}|$$

In the above, $N_{\mathrm{Experiment}(\mathrm{test})}$ and $N_{\mathrm{Ideal}}$ are the number of values over the target threshold in the experiment image and “ideal” image, respectively. It is important to highlight that this is a useful measure of the precision of the target area, but not the accuracy. For this, another metric is required.

- Centroid Difference (CD)

Evaluation of detection accuracy is carried out by assessing the Euclidean difference between the centroid of an “ideal” image polyshape and that of the test image. Assuming the shape has a constant density; the centroid function will take a polyshape and find the exact centre of mass. Using Cartesian coordinates, if the centroid of the “ideal” image is positioned at $i=(i_{1},i_{2})$ and the centroid of the test image is positioned at $t=(t_{1},t_{2})$, then the distance, d(i,t), is calculated in metres using the Pythagorean formula. This is done using the MATLAB pdist function [23].

- Signal-to-Noise Ratio (SNR)

This method of image quantification has basically been derived from the literature of [19]. The SNR is a useful metric in determining how clear any detected inclusion is by providing an assessment of the ratio between the background noise and the desired signal. It is calculated in dBs by using the above mentioned threshold for computing the Polyshape, with the aim of determining the target and background area. The calculations of the SNR are performed through Equation (9):

(9) $$\mathrm{SNR}=10\log_{10}(\frac{Q_{t}-Q_{b}}{D_{b}})\mathrm{dB}$$

In the above equation, $Q_{t}$ and $Q_{b}$ are the mean values of the detected target and background regions, respectively, and $D_{b}$ is the standard deviation of the background.

- Structural Similarity Index Metric (SSIM)

By applying the SSIM method, a value is calculated between 0 and 1, which indicates the structural similarity between two images (with 1 meaning the images are identical) [24]. We proceed by writing the following equation:

(10) $$\mathrm{SSIM}=\frac{\left(2\times \bar{x}\times \bar{y}+C_{1}\right)\left(2\times \sigma_{xy}+C_{2}\right)}{\left(\sigma_{x}^{2}+\sigma_{y}^{2}+C_{2}\right)\left({\bar{x}}^{2}+{\bar{y}}^{2}+C_{1}\right)}$$

where *x* represents the reference image, *y* represents the test image, $\bar{x}$ and $\bar{y}$ are the corresponding mean, $\sigma_{x}$ and $\sigma_{y}$ represent the corresponding variance, $\sigma_{xy}$ is the covariance of the reference and test images. In above equation, $C_{1}$, and $C_{2}$ are small constants. SSIM can be calculated on the basis of two input images using the SSIM function in MATLAB [23]. This will output both a value and a monochrome mapping, which is a useful visual assessment of the structural similarity between the images.

- Signal-to-Clutter Ratio (S/C)

To evaluate the performance of the imaging algorithms in this research, a quantitative metric is used, which is S/C ratio. Clutter comes from undesired scatter from objects within the radar beam that are not targets and has been characterised by several distributions. As the resulting images might possibly contain some clutter even after applying artefact removal procedures, it is applicable to present a parameter with the intention of quantifying and comparing the performance in detection when applying different artefact removal algorithms. Typically, the S/C ratio has been described as the ratio of the maximum brain haemorrhage response to the maximum clutter response. Here, S/C is described as the ratio between the maximum intensity evaluated in the region of the lesion divided by the maximum intensity outside the region of the lesion [25]. This metric evaluates the accuracy and strength of the results and would be highly effective to employ in comparisons.

### 2.4. Description of Phantom

With the aim of validating the MWI system and finding the best method of artefact removal for detection of brain haemorrhagic strokes, a simple phantom is presented. A simple phantom is constructed mimicking the dielectric properties of the human brain, to which a millimetric cylindrically shaped inclusion is applied to emulate a brain haemorrhagic stroke. The two layers mimic: (I) average brain tissues with realistic dielectric properties of white matter and grey matter; and (II) blood inclusion (the inclusion has been placed inside the first layer). There is a dominating region of emulated white and grey matter with a mean conductivity and permittivity of 1.01 S/m and 44, respectively at 1.5 GHz. The emulated blood provides a contrast through its conductivity and permittivity of 1.79 S/m and 60, respectively at 1.5 GHz. The dielectric constant values of both the first layer and the inclusion have been derived from [26]. In order to simulate the dielectric properties of the human brain and haemorrhage tissues, different materials were applied with the purpose of approximating the values described. The mentioned phantom contains different combinations of liquids emulating grey/white matter and blood (brain haemorrhage). Each of the combinations comprises a mixture of deionised water and glycerine at different ratios. Furthermore, choosing the materials was driven by many beneficial aspects such as the stability over long periods of storage time, the low cost of materials, and the ease of access (off-the-shelf). The first layer, which imitates the brain layer, has been fabricated by creating a mixture of deionised water and glycerine with ratios of 60% and 40%, respectively as given in [26]. The inclusion has been mimicked using a combination of 15% glycerine and 85% water [26]. In the mixture proposed, deionised water is employed as the principal substance or source of permittivity, as it shows high dielectric values over a wide bandwidth. Also, a pinch of salt is applied to control the relative permittivity of the compounds. The Epsilon dielectric measurement device (Biox Epsilon Model E100, fabricated at Biox System Company Ltd.) has been used for measuring the electrical properties of the combinations of blood and brain emulating tissue, at room temperature. It should be highlighted that such dielectric properties can be considered representative of brain and an inclusion constituted of blood [26]. A round-bottom cylindrical ABS-Plastic mould with diameter of 110 mm and height of 115 mm has been designed and fabricated to maintain the brain equivalent material. A cylindrically shaped tube with a diameter of 4 mm has been employed to contain an inclusion.

### 2.5. Description of Microwave Imaging (MWI) Device

This research has provided a new application direction for a portable MWI device which has been previously used for breast imaging through phantom measurements [27], and verified in preliminary clinical trials [28]. We have adapted the methods used in this research to detect brain haemorrhage and have produced promising research using the technology for stroke detection.

The MWI device consists of an aluminium cylindrical hub (radius equal to 50 cm) containing two antennas, one transmitting (tx) and one receiving (rx). The hub is internally covered by microwave absorbers, and is equipped with a hole with a cup, allowing the insertion of the object to be imaged. The antennas are installed at the same height, in free space and can rotate around the azimuth in order to collect microwave signals in a multi-bistatic fashion from different angular positions [13].

The tx and rx are connected to a 2-port VNA (S5065, Copper Mountain, Indianapolis, IN, USA). Concerning the transmitting positions, all the experiments have been performed by employing 15 transmitting positions, displaced in 5 triplets centred at 0${}^{{}^{\circ}}$, 72${}^{{}^{\circ}}$, 144${}^{{}^{\circ}}$, 216${}^{{}^{\circ}}$, and 288${}^{{}^{\circ}}$. In each triplet, the transmitting positions are displaced by 4.5${}^{{}^{\circ}}$. For each transmitting position, we recorded the complex S21 at $N_{\mathrm{PT}}$ = 80 receiving positions, uniformly displaced along a circular surface having radius $a_{0}$ = 7 cm (the receiving positions are denoted with the index *np* = 1, 2, ..., 80, and the transmitting positions with the index *m* = 1, 2, ..., 15). Specifically, we measure complex S21 at the points $rx_{\mathrm{np}}$ ≡ $\left(a_{0};\phi_{\mathrm{np}}\right)$ ≡ ${\vec{\rho }}_{\mathrm{np}}$, displaced along a circular surface having radius $a_{0}$. For each transmitting and receiving position, the complex S21 is collected from 1 to 1.5 GHz, as this band has been demonstrated as being ideal and optimal for brain imaging [1], with 5 MHz sampling.

**Figure 1:**
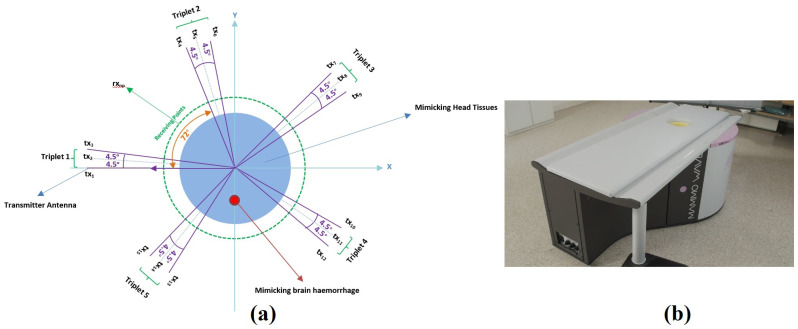
(**a**) Pictorial view of the Microwave Imaging (MWI) device configuration including the human brain and stroke phantom, (**b**) The MWI device.

Figure 1a shows a sketch of the configuration of the MWI device, where the light green dashed circle indicates the perimeter where the receiving antenna can be moved circularly, in order to receive the signals from different positions. The transmitting antennas positions are displaced along a circular surface having radius of 35 cm. The brain phantom and inclusion (imitating blood) are presented in light blue and red respectively. Figure 1b shows the MWI device.

## 3. Results

In this paper, our aim is to prove that our proposed method has the potential to successfully detect haemorrhagic stroke. Another purpose of this paper is to discover the best technique of artefact removal, specifically when it comes to the detection of brain haemorrhage. To this end, the experiments have been executed by considering the combination of five transmitting position triplets. The images are obtained after applying five different signal pre-processing methods, functioning as the artefact removal procedures. The results are presented for an inclusion at four different positions of 0${}^{{}^{\circ}}$, 90${}^{{}^{\circ}}$, 180${}^{{}^{\circ}}$, and 270${}^{{}^{\circ}}$.

**Figure 2:**
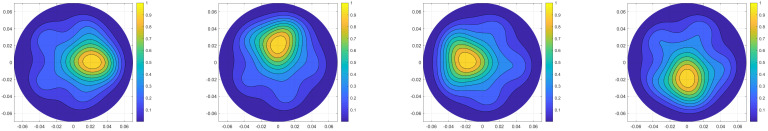
Ideal image generated by subtracting data of the phantom with no inclusion from the one with an inclusion (positioned at 0${}^{{}^{\circ}}$, 90${}^{{}^{\circ}}$, 180${}^{{}^{\circ}}$ and 270${}^{{}^{\circ}}$), respectively.

Figure 2 illustrates the final normalised microwave image of such phantom with an inclusion. In this and the following Figures, both x and y axes are in metres, while the intensity has an arbitrary unit. We apply the expression of the “no target” and “target” scenarios ($S21_{\mathrm{np},tx_{m}}^{\mathrm{rcstr},\mathrm{target}}-S21_{\mathrm{np},tx_{m}}^{\mathrm{rcstr},\mathrm{notarget}}$) to the recorded S21 before performing imaging, in order to construct a reference for comparison with the resulting images from using other artefact removal methods. Figure 2 is used as an “ideal” image for reference and comparisons.

**Figure 3:**
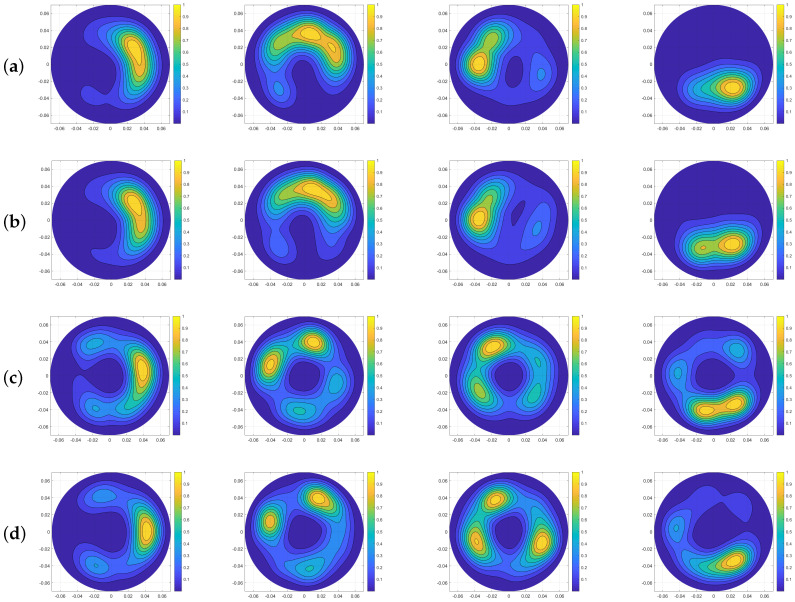
Combination of 5 transmitting position triplets using Rotation Subtraction (RS), Local Average Subtraction (LAS), Differential Symmetric Receiver Type (DSR), and Summed Symmetric Differential (SSD) artefact removal methods for inclusion positioned at 0${}^{{}^{\circ}}$, 90${}^{{}^{\circ}}$, 180${}^{{}^{\circ}}$, and 270${}^{{}^{\circ}}$, respectively. Microwave images obtained using (**a**) RS, (**b**) LAS, (**c**) DSR, and (**d**) SSD artefact removal methods.

Figure 3 shows the combination of 5 transmitting position triplets for inclusion at 0${}^{{}^{\circ}}$, 90${}^{{}^{\circ}}$, 180${}^{{}^{\circ}}$, and 270${}^{{}^{\circ}}$. The images are obtained after employing the RS (between two triplet positions), LAS, DSR type, and SSD methods, functioning as the artefact removal procedures.

**Figure 4:**
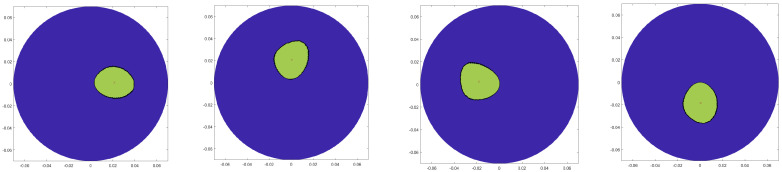
Ideal polyshape image using measured data for inclusion at 0${}^{{}^{\circ}}$, 90${}^{{}^{\circ}}$, 180${}^{{}^{\circ}}$, and 270${}^{{}^{\circ}}$, respectively.

The imaging performance has been analysed using image quantification. With the aim of evaluating the impact of transmitting positions in achieving detection, six different metrics for the obtained final images (from the combination of 5 transmitting position triplets) have been calculated. Figure 4 shows the images of the “ideal” polyshapes, constructed using ideal results obtained through the “no target” and “target” scenarios and applying polyshape functions.

**Figure 5:**
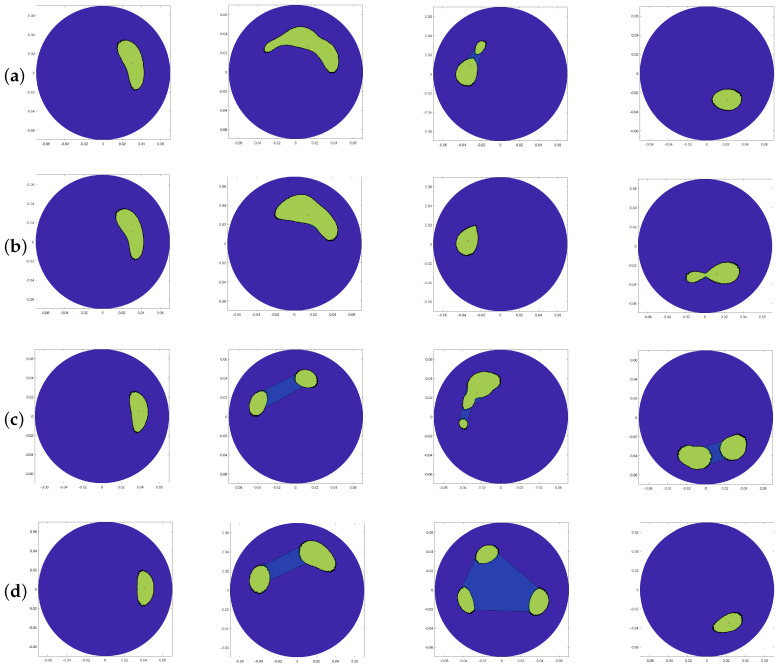
Resulting polyshape images obtained using measured data after performing (**a**) RS, (**b**) LAS, (**c**) DSR, and (**d**) SSD artefact removal methods for inclusion at 0${}^{{}^{\circ}}$, 90${}^{{}^{\circ}}$, 180${}^{{}^{\circ}}$, and 270${}^{{}^{\circ}}$, respectively.

Figure 5 indicates the resulting polyshape images, obtained using RS, LAS, DSR, and SSD artefact removal methods. Four different locations of the inclusion have been considered.

The correspondent S/C is given in Table 1 for all artefact removal methods. The methods are expressed in abbreviated form. In this table both the S/C values for the inclusion positioned at 0${}^{{}^{\circ}}$, 90${}^{{}^{\circ}}$, 180${}^{{}^{\circ}}$, and 270${}^{{}^{\circ}}$ and the average S/C values of different inclusion positions have been presented.

Table 2 provides a valid comparison between different artefact removal methods for the inclusion positioned at 0${}^{{}^{\circ}}$, 90${}^{{}^{\circ}}$, 180${}^{{}^{\circ}}$, and 270${}^{{}^{\circ}}$. It is worth pointing out that for the sake of simplicity, the valid comparison is made using the average values of different inclusion positions in this table. In addition, the values are not absolute numbers and are used to indicate to what extent the “ideal” image is similar to the images obtained through different artefact removal methods. The methods and metrics are expressed in abbreviated form.

It is important to mention that the LAS method will be considered for each triplet individually. The greater the values of SNR, SSIM, S/C and lower ArD and CD are, the higher the performance of the method is. Ideally, the SSIM should be equal to one and the CD, and ArD should be equal to zero.

## 4. Discussion

The results shown in the previous section confirm the potential of the procedure in providing reliable outcomes, as it is capable of detecting a target (emulating brain haemorrhage) placed at four different positions. Through this research, an “ideal/reference” image is used to compare the proposed artefact removal methods by employing six different image quality metrics. These metrics were proposed in order to perform a quantification of detection accuracy and precision. The procedure was able to detect the target inside a two-layer brain phantom.

Our previous work in [12] has confirmed that the HP based procedure permits detection (in an anechoic chamber) of strong scatterers and can distinguish between different tissues in the final image. Following on from this initial exposition, there is a medical requirement for a portable brain stroke imaging device. Since the results coming from measurements inside the anechoic chamber are not representative of a realistic medical device scenario, we have developed a novel procedure using the dedicated imaging prototype. From the obtained results, we can conclude that the LAS and RS are the best approaches to remove artefacts, i.e., transmitter image and the reflections of the layers. Even so, it may be the case that even beyond artefact removal, the residual clutter masks the inclusion. Residual clutter is due to the imperfect cancellation of the transmitting antenna, inappropriate cancellation of the first layer reflection or even due to multiple reflections occurring inside the phantom that cannot be cancelled completely. Based on the achieved result, the highest values of SNR, SSIM, and S/C and the lowest values of CD and ArD are obtained when employing the RS and LAS artefact removal methods. More specifically, as seen in Table 1 and Table 2, LAS has the highest values of SSIM and average S/C, which are 0.87 and 2.03 (linear scale), respectively. Meanwhile, the maximum SNR is allocated to the RS method with 5.06. It has also been shown that the lowest values of both CD and ArD belong to LAS method with 0.014 m and 0.16, respectively. The average S/C (linear) calculated for the “ideal” artefact removal method at the frequency of 1–1.5 GHz when considering different positions of inclusion is equal to 2.62. When employing different artefact removal methods to the measured data, a decrease in S/C can be observed, with a minimum average value of 1.41 (linear scale) related to SSD method, as given in Table 2. It follows that the use of the LAS and RS artefact removal methods, in a realistic scenario, implies a S/C reduction of less than 3 dB with respect to the ideal case of performing artefact removal through subtraction between the “no target” scenario and the “target” scenario. Also, it is important to highlight that the results show that the position of the inclusion can vastly influence the resulting image, with every artefact removal method exhibiting differences in image quality, when the inclusion was moved.

When the inclusion is positioned at 90${}^{{}^{\circ}}$ when applying the DSR and SSD methods, all images show a symmetric split inclusion image. This might be due to the direct fields and the fields reflected by the first layer. The same thing happens at position 180${}^{{}^{\circ}}$ when using the RS, DSR and SSD artefact removal methods, and at position 270${}^{{}^{\circ}}$ when applying the LAS and DSR methods, as shown in Figure 5. Ultimately, by visually comparing the resulting polyshape with “ideal” images and their dedicated metrics, it has been concluded that the RS and LAS artefact removal methods are the best techniques for eliminating the artefacts. The results shown in this paper, achieved through measurements using an MWI device, pave the way towards the validation of a reliable artefact removal method and use of a low complexity, portable MWI device for haemorrhagic brain stroke detection, where antennas operate in free-space and which has the potential for pre-hospital use.

## 5. Conclusions and Future Work

In this manuscript, a set of artefact removal algorithms are provided for comparison using a variety of suitable performance metrics. Specifically, we presented an experimental assessment of different artefact removal methods using an MWI device based on an HP algorithm for brain haemorrhagic stroke detection. This procedure was able to provide images indicating the target which mimics brain haemorrhage (an inclusion) positioned at four different locations inside a dedicated cylindrical phantom, based on the differences in dielectric properties. However, none of the artefact removal methods require a cylindrical geometry, thus they can be used for others geometries too (including realistic head).

Our findings on artefact removal algorithms have been derived using a 2D configuration and a multi-bistatic arrangement, but they apply also to 3D configurations and to arrangements other than the multi-bistatic one.

The results shown in this paper, achieved through measurements using an MWI device, pave the way towards the validation of a reliable artefact removal method and use of a low complexity, portable MWI device for haemorrhagic brain stroke detection, where antennas operate in free-space (coupled through a VNA). The MWI device is safe (no X-rays) and portable.

Different shape/size of the inclusions may affect artefact removal methods performances; in this context, a dedicated investigation should be performed to characterise such performances with respect to inclusions shape/size. Finally, a further step of the procedure development and validation will involve distinguishing between different sorts of stroke (e.g., the brain ischemic and haemorrhagic stroke).

## Figures and Tables

**Table 1:** Signal-to-Clutter ratio (S/C) (linear) for the frequency band between 1 to 1.5 GHz (using measurements data with MWI device and RS, LAS, DSR, and SSD artefact removal methods).

Positions	RS	LAS	DSR	SSD	Ideal
Position 0${}^{{}^{\circ}}$	1.92	1.93	1.74	1.75	2.64
Position 90${}^{{}^{\circ}}$	1.87	2.45	1.02	1.13	2.59
Position 180${}^{{}^{\circ}}$	1.84	1.92	1.33	1.00	2.65
Position 270${}^{{}^{\circ}}$	2.01	1.81	1.76	1.75	2.63
Average	1.91	2.03	1.46	1.41	2.62

**Table 2:** Artefact removal comparison.

Artefact Removal Methods	ArD	CD	SNR	SSIM
RS	0.17	0.016	5.06	0.86
LAS	0.16	0.014	4.94	0.87
DSR	0.21	0.020	4.53	0.84
SSD	0.19	0.018	4.03	0.78
